# Photoelectrochemistry of Redox-Active Self-Assembled Monolayers Formed on n-Si/Au Nanoparticle Photoelectrodes

**DOI:** 10.1021/acs.langmuir.4c01751

**Published:** 2024-08-07

**Authors:** Kayla M. Mancini, Yousef Khatib, Lauren Shahine, Glen D. O’Neil

**Affiliations:** † Department of Chemistry and Biochemistry, Montclair State University, Montclair, New Jersey 07043, United States; ‡ Sokol Institute for Pharmaceutical Life Sciences, Montclair State University, Montclair, New Jersey 07043, United States

## Abstract

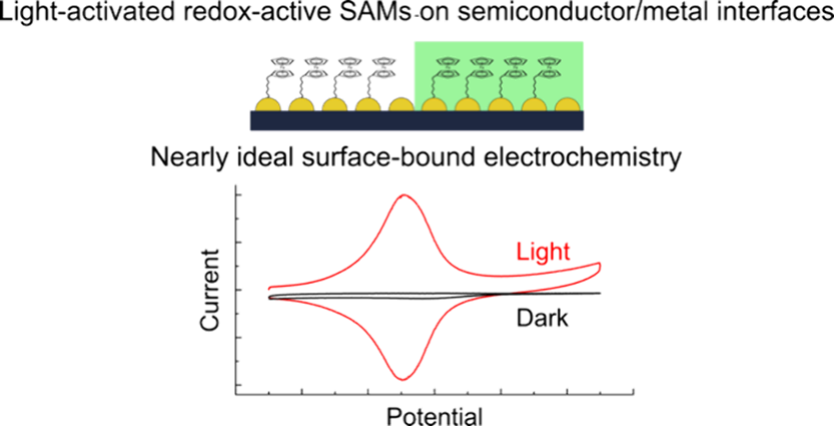

Controlling the chemistry of the electrode–solution interface is critically important for applications in sensing, energy storage, corrosion prevention, molecular electronics, and surface patterning. While numerous methods of chemically modifying electrodes exist, self-assembled monolayers (SAMs) containing redox-active moieties are particularly important because they are easy to prepare, have well-defined interfaces, and can exhibit textbook photoelectrochemistry. Here, we investigate the photoelectrochemistry of redox-active SAMs on semiconductor/metal interfaces, where the SAM is attached to the metal site instead of the semiconductor. *n*-Si/Au photoelectrodes were fabricated using a benchtop electrodeposition procedure and subsequently modified by immersion in aqueous solutions of (ferrocenyl)hexanethiol and mercaptohexanol. We explored the relevant preparation conditions, finding that after optimization, we were able to obtain canonical cyclic voltammetry for a surface-bound redox molecule that could be turned on and off using light. We then characterized the optimized electrodes under varying illumination intensities, finding that the heterogeneous electron transfer kinetics improved under higher illumination intensities. These results lay the foundation for future studies of semiconductor/metal/molecule interfaces relevant to sensing and electrocatalysis.

## Introduction

Chemical control over the electrode/solution interface is crucial for designing electrodes with variable properties.^1,2^ Redox-active self-assembled monolayers (SAMs) are widely used to control the electrode/solution interface because they offer well-ordered, precise control of the chemical environment of the redox molecule.^3,4^ These modified electrode surfaces have a broad range of applications in sensing,^5−10^ energy storage and conversion,^11−13^ corrosion prevention,^14^ molecular electronics,^15−19^ surface patterning,^20,21^ and for tuning interfacial energetics.^22^ Redox-active SAMs are also useful for testing fundamental theories of electron transfer in both metals^23−26^ and semiconductors.^27−31^ Consequently, there are numerous approaches to understanding (and modeling) the voltammetry of redox molecules adsorbed on surfaces, which are useful for extracting kinetic and thermodynamic parameters from experiments.^30−35^

While modifying metallic, and in particular Au, electrodes with redox-active SAMs can be accomplished on the benchtop, modification of semiconductor, particularly Si, surfaces is more challenging.^36^ However, modification of Si with redox-active SAMs is particularly important for the electronics industry, where the need for miniaturization is driving advances in molecular-level interface control.^37^ The main challenge in forming well-ordered, electrochemically well-behaved monolayers on Si is preventing oxide growth, necessitating multistep syntheses in air- and water-free environments.^38,39^ Modifying Si photoelectrodes with metal films, nanoparticles, or 2D materials is another common approach for controlling the energetics and chemistry of the Si interface.^40,41^ These types of interfaces are widely used as diodes in the electronics industry and as water-splitting photoelectrodes for energy storage applications.^42−44^ For electrochemical applications, an advantage of modifying semiconductor surfaces with metals is that the electrochemical properties of the metal are largely conserved, essentially creating metallic electrodes that can be turned on/off with light. One drawback of this approach is the lack of direct chemical control over the metal surfaces, which often change dramatically during electrolysis.^45^

In this study, we investigate the photoelectrochemistry of redox-active SAMs on semiconductor/metal junctions, where the chemical modification occurs at the metal site instead of the semiconductor. To the best of our knowledge, there are no previous reports of redox-active SAMs formed on semiconductor/metal interfaces, although there are numerous examples of semiconductor/molecule/metal junctions in the literature (reviewed in ref (16)). As a first demonstration, we modified *n*-Si electrodes with electrodeposited Au NPs, onto which we formed a redox-active SAM consisting of (ferrocenyl)hexanethiol (FcHT) and mercaptohexanol (MCH). We characterized the electrodes using atomic force microscopy (AFM), X-ray photoelectron spectroscopy (XPS), electrochemical impedance spectroscopy (EIS), and cyclic voltammetry (CV). The important new result is that under optimized conditions, the photoelectrochemistry of the *n*-Si/Au/FcHT photoelectrodes showed fast electron transfer and near-ideal figures of merit for surface-bound redox molecules under illumination, but no redox activity in the dark. We explored how the preparation conditions (i.e., FcHT concentration, metal morphology, and SAM deposition time) affect the observed photoelectrochemistry. Finally, we measured how illumination intensity changes the voltammetry, and in particular the electron transfer kinetics, of the redox-active SAM. This work is part of a larger effort within our group to expand the scope and application of semiconductor/metal light-addressable electrochemical (LAE) sensors; attainment of that goal necessitates understanding the photoelectrochemistry of tethered redox species to these interfaces. This work lays the foundation for future studies in sensing and electrocatalysis with modified semiconductor/metal photoelectrodes.

## Experimental Section

### Materials and Solutions

6-(Ferrocenyl)hexanethiol (FcHT) and 6-mercaptohexanol (MCH; 97%) were purchased from Sigma-Aldrich. Phosphate-buffered saline (PBS) reagent tablets, KCl, K_2_SO_4_, HClO_4_, and H_2_SO_4_ were from Fisher and were of ACS Reagent grade. Ferrocene methanol (FcMeOH; 97%) was obtained from Acros Organics. Ammonium fluoride (NH_4_F; 40% m/m, semiconductor grade) was from Honeywell. Hydrogen tetrachloroaurate(III)trihydrate (HAuCl_4_; Au 49% min, crystalline) was purchased from Alfa Aesar. All solutions were prepared using 18.2 MΩ·cm DI water (Millipore Simplicity).

### Electrode Preparation

The photoelectrodes used in this study were prepared using n-type Si (100) from Pure Wafer (San Jose, California, USA). The n-type wafers were doped with phosphorus (resistivity 1–5 Ω·cm). Control experiments were conducted with p^+^-Si (100) wafers (resistivity <0.005 Ω·cm) that were highly doped with boron. Si wafers were diced into ∼1 cm^2^ samples by scoring the unpolished side with a diamond-tipped pen and breaking the wafer along the scratch line. Each sample was cleaned with Piranha solution (a 3:1 v/v mixture of concentrated H_2_SO_4_ to 30% H_2_O_2_) for 30 min at 105 °C. *Caution: Piranha solution is highly corrosive and reacts dangerously with organics.* The samples were thoroughly rinsed with DI water before being submerged in a 40% NH_4_F solution (previously deoxygenated with Ar for 30 min) for 10 min to remove the oxide on both sides of the sample. H-termination of the Si was confirmed by placing a small droplet of water on the surface to confirm hydrophobicity. Ohmic back connections were prepared by contacting a Cu wire using indium solder. The back contacts were insulated by sealing the entire assembly in 3 M Electroplater’s tape (3 M 470), with a 3 mm-diameter circular opening that allowed exposure of the polished front Si surface to the electrolyte. This protocol was performed in batches of ≈20 electrodes that were stored in the dark until future use.

The exposed Si surface was etched in a deaerated 40% NH_4_F solution for 10 min at room temperature to remove the native oxide. The electrode was rinsed with copious amounts of DI water prior to electrodeposition. Au NPs were electrodeposited onto the polished front surface of the Si in order to establish a semiconductor/metal junction and increase the electronic coupling between the semiconductor and redox species, using a modified procedure described by Allongue et al.^46^ To minimize oxidation, the electrode was biased to −1.945 V vs SCE before being dipped into the electrodeposition solution. A glassy carbon rod was used as the counter electrode during electrodeposition to prevent contamination of the n-Si with Pt. The electrodeposition solution consists of 0.2 mM HAuCl_4_, 1 mM KCl, 0.1 M K_2_SO_4_, and 1 mM H_2_SO_4_. Electrodeposition was carried out with the room lights on using a deposition time of 5 min. After electrodeposition, the samples were rinsed with copious amounts of DI water.

Electrodes were modified with a self-assembled monolayer consisting of FcHT by immersing electrodes in an aqueous FcHT solution in 0.02 M PBS for 60 min. Electrodes were rinsed with copious amounts of DI water and then were immediately incubated in 30 mM MCH solution for 90 min to passivate unbound electrode surface. Incubations were performed in sealed 50 mL conical tubes (Falcon).

### Photoelectrochemical Measurements

Electrochemical measurements were performed using a CH Instruments 760E potentiostat. All electrochemical measurements were carried out in a 30 mL electrochemical cell with a borosilicate glass window with a three-electrode configuration. A saturated calomel electrode (SCE) served as the reference and a glassy carbon rod as the counter. The semiconductor was illuminated with either a Boling Super LED (amazon.com) or a white-light LED from AmScope. The intensity of the white light was measured to be ≈80 mW/cm^2^. All optical components were housed inside an optical enclosure purchased from Thorlabs.

CV measurements were carried out under direct illumination and in the dark in an electrolyte containing 0.1 M HClO_4_ over a potential range from approximately −0.4 to 0.4 V vs SCE. EIS for Mott–Schottky measurements were carried out in the dark in 0.1 M HClO_4_ over a potential range, −1 to 0 V vs SCE. When required, light intensity was modulated using a series of neutral density filters from ThorLabs (NEK01S) and calibrated using a ThorLabs PM161-121 USB power meter. In all the photoelectrochemical measurements presented herein (unless otherwise stated), the illumination intensity (≤80 mW cm^–2^) is sufficiently high to have the response of the electrode be governed by the electrochemistry rather than carrier generation/transport. Reproducibility was evaluated by performing all measurements with multiple independently prepared samples (*n* ≥ 3). The reported figures of merit are reported as the mean ± uncertainty, which was represented by the standard deviation (or 95% confidence interval) of the mean. Where appropriate, statistical comparisons were made using either a Student’s *t* test or ANOVA with Bonferroni correction. We used the Peak Analyzer toolkit in OriginPro 8.5 for determining FWHM values and for performing deconvolution of the voltammograms in Figures 5 and 6.

### Physical Characterization

We performed AFM using a Bruker Dimension Icon AFM to characterize the surface morphology of the electrodeposited Au NPs. The images were collected in ScanAsyst mode using a SCANASYST-AIR-HPI probe (7 nm radius, *k* = 0.25 N/m, *f* = 55 kHz). The imaging rate was 0.5 Hz with a resolution of 512 samples/line. All images were 3 μm × 3 μm, making each pixel ≈6 nm, similar to the probe radius. XPS was performed on a Thermo Scientific K-Alpha XPS at the Surface Analysis Facility at the University of Delaware. Survey scans were conducted over the range 0 to 1360 eV. Single-element scans for Au, Si, Fe, S, O, and C were also performed.

## Results and Discussion

The goal of this study was to characterize the photoelectrochemistry of semiconductor/metal/molecule interfaces formed by depositing redox-active SAMs on the metal surface of a semiconductor/metal interface. While the modification of metal and silicon surfaces with redox-active SAMs is common in the literature, we were unable to find any examples of redox-active SAMs formed on semiconductor/metal interfaces.

### Fabrication of n-Si/Au/FcHT Interfaces

**Figure 1:**
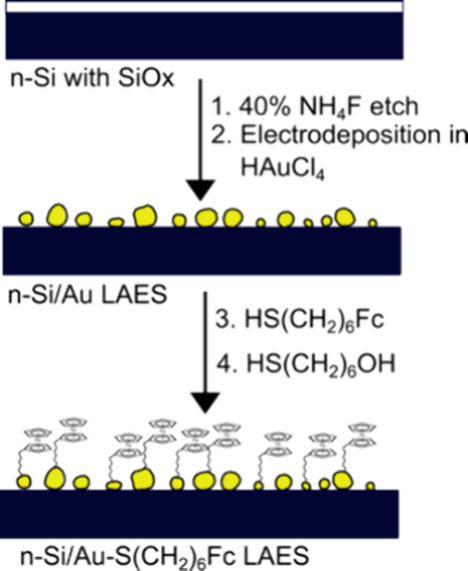
A schematic showing the preparation of the self-assembled monolayers on n-Si/Au junctions.

We prepared n-Si/Au electrodes by electrodeposition at −1.945 V vs SCE for 5 min from an electrolyte containing HAuCl_4_, 0.1 M K_2_SO_4_, 1 mM KCl, and 1 mM H_2_SO_4_ (see Experimental Section for details).^46−49^ These electrodes were subsequently modified with redox-active SAMs consisting of FcHT and MCH (Figure 1) by incubating the electrodes in an aqueous phosphate buffer solution containing FcHT for a fixed time to allow the formation of the SAM.^50^ The electrodes were thoroughly rinsed and then submerged in a 30 mM solution of MCH to passivate unreacted Au sites and form a mixed monolayer. We hypothesized that due to sulfur’s higher affinity for Au than Si, the thiolated redox molecules would selectively bind to the Au surfaces via a gold–thiol bond. We systematically studied the effect of FcHT concentration, SAM incubation time, and morphology of the Au layer to understand how each of these factors contributes to the observed voltammetry.

### Physical Characterization of n-Si/Au NP/SAM Interfaces

**Figure 2:**
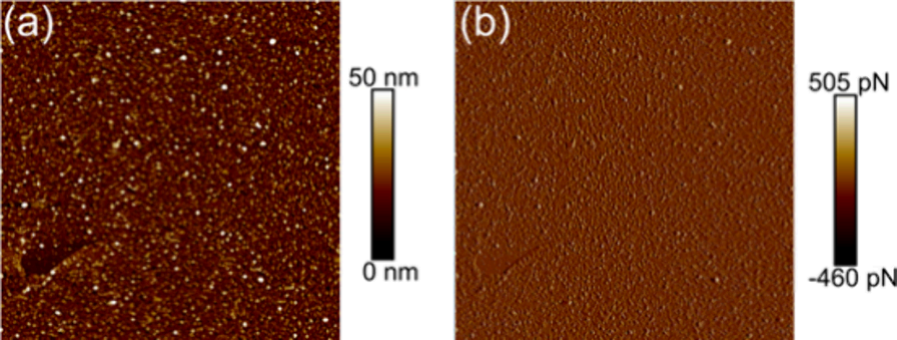
AFM characterization of the n-Si/Au photoelectrodes prepared by electrodepositing Au from an electrolyte containing 0.2 mM HAuCl_4_, 0.1 M K_2_SO4, 1 mM H_2_SO_4_, and 1 mM KCl in (a) height mode and (b) peak force error mode. The measurements in part (b) are not quantitative and are only used to help visualize the contrast of the height image in part (a). AFMs were acquired over 3 μm × 3 μm in ScanAsyst mode using SCANASYST-AIR-HPI probes (7 nm radius, *k* = 0.25 N/m, *f* = 55 kHz).

We characterized the morphology and elemental composition of n-Si/Au and n-Si/Au/FcHT interfaces using AFM and XPS, respectively. Figure 2a shows a representative AFM height image (3 μm × 3 μm) of an n-Si/Au sample prepared as described above. The images presented in Figure 2 are representative of multiple images acquired at multiple locations on three separate samples. However, even with multiple imaging locations on multiple samples, we are only characterizing ≈4 × 10^–4^% of the electrode surfaces. The AFM height image confirms that the electrodeposition procedure produces heterogeneous n-Si/Au surfaces that are largely composed of small, closely spaced NPs. Figure 2b shows the corresponding AFM peak force error image, which is a measure of the peak force deflection during ScanAsyst imaging and is caused by different tip–substrate interactions between dissimilar materials. The measurements in Figure 2b are not quantitative and are only used to help visualize the contrast of the height image in Figure 2a.

**Figure 3:**
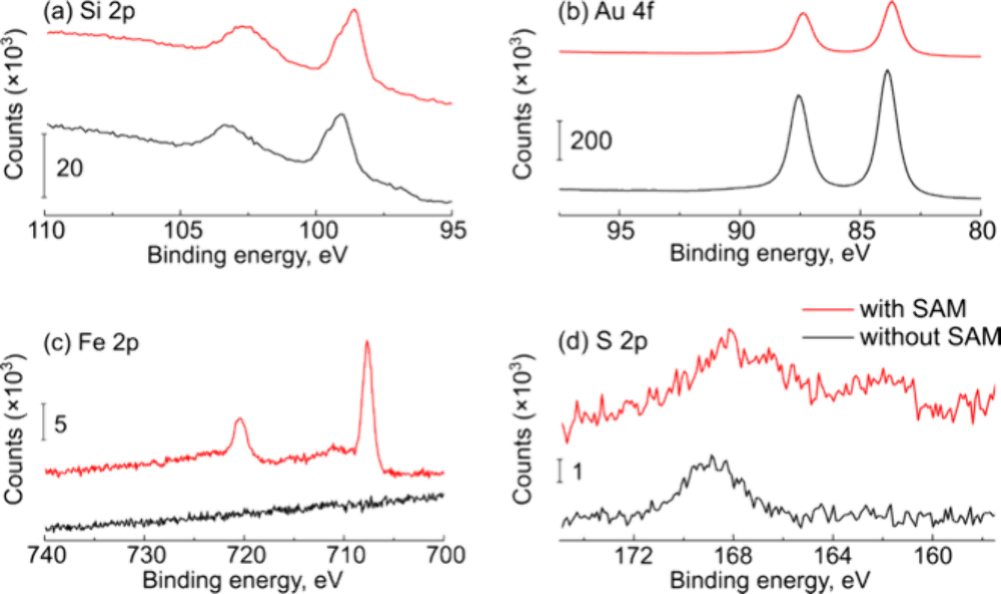
Elemental characterization of n-Si/Au and n-Si/Au/FcHT photoelectrodes using XPS. (a) Si 2p region; (b) Au 4f region; (c) Fe 2p region; (d) S 2p region. The black traces are n-Si/Au electrodes, and the red traces are n-Si/Au/FcHT-MCH electrodes.

We performed XPS to confirm that the FcHT was successfully attached to the Au NP. Figure 3 shows single-element XPS data for n-Si/Au (black traces) and n-Si/Au/FcHT (red traces) interfaces. Survey scans (Figure S1) and single-element scans for the C 1s and O 1s (Figure S2) are presented in the Supporting Information. Figure 3a shows Si 2p XPS spectra, with two peaks evident. The peak at ≈99 eV corresponds to elemental Si, and the broad peak between 102 and 105 eV corresponds to oxidized silicon species.^51^ It is unknown if the oxide forms during electrodeposition or if the Si was oxidized in the lab ambient between preparation and analysis (approximately 5 days). However, the presence of the oxide does not seem to dramatically impact the ability of the electrodes to perform fast electron transfer (*vide infra*). Figure 3b shows the Au 4f_5/2_ (≈88 eV) and 4f_7/2_ (≈84 eV) spectra, consistent with the formation of gold nanoparticle films. The intensity of both gold spectral peaks is attenuated after the formation of the monolayer, consistent with SAM formation on Au surfaces. The ratio of the peak intensities of the 4f_7/2_/4f_5/2_ is nearly identical for both systems (1.23 for n-Si/Au samples and 1.25 for n-Si/Au/FcHT samples), suggesting a similar average oxidation state for the gold particles before and after SAM formation.^52^ The XPS suggests that all of the Au is present as Au(0), consistent with other Au-SAM interfaces.^52^Figure 3c shows Fe 2p_3/2_ and 2p_1/2_ peaks at ≈708 and ≈721 eV, respectively. These peaks are associated with the Fe^2+^ oxidation state of the neutral ferrocene molecule.^53^ Importantly, the n-Si/Au samples without a SAM showed no evidence of Fe in the XPS. Figure 3d shows the high-resolution S 2p span with peaks at ≈162 and ≈168 eV. Note that the intensity of these peaks is very low compared with the other elements studied. The peak at ≈162 eV is associated with bound thiols, while the peak at ≈168 is suggestive of oxidized sulfur species.^54^ We observed the peak at ≈168 eV in the samples prepared without a monolayer. Given that the electrodeposition bath contains significant amounts of sulfate (>0.1 M), we tentatively assign this peak to residual sulfate present in the Au layer. Ion incorporation into thin films of other metals has been previously demonstrated.^55^ Importantly, there is no evidence of unbound thiol, which would be present at ≈164 eV.^54^ The lack of evidence for unbound thiol suggests that a monolayer of FcHT and MCH is formed under the preparation conditions. Taken together, the data in Figures 2 and 3 show that the electrodeposited nanoparticles are gold and that FcHT/MCH can be attached to the n-Si/Au surface through the formation of gold–thiol bonds.

### Characterization of the Junction Energetics of n-Si/Au/FcHT Electrodes

Characterizing the energetics (i.e., the positions of the flat-band potential (*E*_fb_), the conduction edge (*E*_cb_), and the valence band edge (*E*_vb_)) of the n-Si/Au and n-Si/Au/FcHT junctions is an important first step toward understanding the photoelectrochemistry of the interfaces, because it determines the applied potential range where n-Si will be depleted of minority carriers.^56^ When the semiconductor is depleted of minority carriers, the electrochemical reaction can be turned on/off using light. We compared n-Si/Au electrodes with and without an FcHT monolayer to understand if the presence of the monolayer influenced the band energetics. Figure S3 in the Supporting Information shows representative plots of reciprocal square capacitance versus the applied potential for n-Si/Au and n-Si/Au/FcHT interfaces submerged in a deaerated 0.1 M HClO_4_ solution. Qualitatively, the two sets of data are nearly identical. The calculations for extracting the various parameters is detailed in the Supporting Information, Section S2, and a table summarizing the results is presented in Table 1. We estimated *E*_fb_ of the n-Si/Au and n-Si/Au/FcHT samples to be −0.36(±0.05) and −0.37(±0.01) V vs SCE, respectively (all data reported as mean ± *s*; *n* ≥ 3). Using the slope of the linear portion of the *C*^–2^ vs *E* plot, we estimated the charge carrier densities, *N*_d_, to be 9(±3) × 10^14^ and 7(±4) × 10^14^ cm^–3^ for n-Si/Au and n-Si/Au/FcHT samples, respectively. These carrier concentrations correspond to a wafer resistivity of ≈5 Ω·cm (estimated using reference^57^), which is consistent with the manufacturer’s stated value (1–5 Ω·cm). We estimated *E*_cb_ to be −0.62(±0.05) and −0.64(±0.02) V and *E*_vb_ to be 0.48(±0.05) and 0.46(±0.02) for samples without and with the SAM, respectively. None of the values are statistically different at the 95% confidence limit (two-tailed Student's *t* test).

**Table 1:** Summary of the MS Figures of Merit^a^

sample	*E*_fb_ (V) vs SCE	*N*_d_ (cm^–3^)	*E*_vb_ (V) vs SCE	*E*_cb_ (V) vs SCE
n-Si/Au	–0.36(±0.05)	9(±3) × 10^14^	0.48(±0.05)	–0.62(±0.05)
n-Si/Au/FcHT	–0.37(±0.01)	7(±4) × 10^14^	0.46(±0.02)	–0.64(±0.02)

a Measurements are presented as the average of three individually prepared samples with the uncertainty represented by one standard deviation.

The data above provide an estimate of the band energetics of the semiconductor/metal and semiconductor/metal/molecule interfaces. The first important result is that FcHT binding does not appear to influence the energetics of the semiconductor/metal junction; each figure of merit is statistically similar when comparing n-Si/Au and n-Si/Au/FcHT interfaces. This result is surprising given that Fc-containing SAMs are known to alter the work function of metallic Au films.^58^ We suspect that the consistent *E*_fb_, *E*_vb_, and *E*_cb_ values are observed because the potential of the redox molecule is close to the valence band edge.^59^ The second important result is that for all applied potentials greater than *E*_fb_ (approximately −0.36 V), n-Si will be in depletion (and hence photoactive). Given that *E*^0^ is approximately 0.3 V for FcHT on Au, the electrochemistry should be light-addressable. The third important observation is that *E*_fb_, *E*_cb_, and *E*_vb_ are all significantly more positive than our previously reported n-Si/Au photoelectrodes.^48,49^ In our previous studies, we characterized the band energetics in neutral (or near-neutral) pH solutions containing 0.1 M KNO_3_ and found samples to have *E*_fb_ of approximately −0.7 V vs SCE.^48,49,59^ We suspect that the shift in flat-band potential may be caused by a shifting of the Si bands with pH, since the Si band positions shift toward more positive values in acidic solutions.^60^

### Electrochemical Behavior of n-Si/Au/FcHT Electrodes

As described in detail elsewhere,^4,61^ a SAM containing a well-behaved redox-active species should display several distinct characteristics: (i) the oxidation and reduction waves should have Gaussian shapes with peak currents that are directly dependent on the scan rate; (ii) there should be no difference in the anodic and cathodic peak potentials (i.e.; Δ*E*_p_ = *E*_p,a_ – *E*_p,c_ ≈ 0 mV); (iii) the full width at half-maximum of the peaks should be 90.6 mV; (iv) only one wave should be observed in the forward and reverse scans. In general, these characteristics can be observed when the redox-active sites do not interact with each other in the SAM and when the heterogeneous electron transfer (HET) kinetics are fast compared with the CV scan rate.

**Figure 4:**
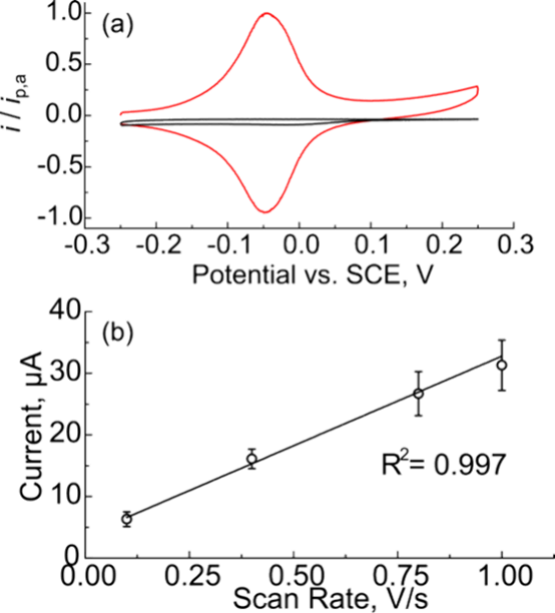
(a) CVs of n-Si/Au/FcHT electrodes in 0.1 M HClO_4_ at 0.1 V s^–1^ in the absence (black trace) and presence (red trace) of ≈80 mW cm^–2^ illumination. (b) Plot of peak current versus scan rate for n-Si/Au/FcHT electrodes. The data points and error bars represent the mean and the 95% confidence interval, respectively, of four independent samples.

We used CV to characterize the electrochemical behavior of n-Si/Au/FcHT electrodes in the presence and absence of light to determine if the electrochemistry was activated by light. Figure 4a shows CVs of a n-Si/Au/FcHT electrode immersed in a 0.1 M HClO_4_ solution in the absence and presence of ≈80 mW cm^–2^ white light at 0.1 V s^–1^. CVs collected in the dark (black trace) show negligible current, which is consistent with n-Si being in depletion. Under illumination, electron–hole pairs are generated within the Si. The holes are transported to the Au surface due to band bending induced by the metal particles and react with the FcHT bound to the Au surface. The generation, transport, and collection of photogenerated carriers effectively “turns on” the electrochemical response of the n-Si/Au/FcHT electrodes, as seen in the red trace in Figure 4a. Qualitatively, the illuminated CV shows characteristics of a surface-bound redox species, notably Gaussian peak shapes with very little separation between anodic and cathodic peak potentials. Δ*E*_p_ between the anodic and cathodic scans was ≈2 mV for this sample at 0.1 V s^–1^, which is very close to the theoretical value of 0 mV and suggests fast, near-reversible charge transfer from the n-Si/Au to the surface-bound Fc. The FWHM value of the anodic peak was 93 mV at 0.1 V s^–1^, which is also consistent with fast kinetics. The position of the redox wave is approximately −0.05 V vs SCE, less than the standard reduction potential of FcHT on Au (≈0.3 V vs SCE). The cathodic shift in potential is consistent with the semiconductor being in depletion and is caused by energy from the absorbed light shifting the electrode potential to more anodic values than those applied by the potentiostat.

In some samples (estimated as ≈1/3 of hundreds of samples fabricated over more than 6 months), we observe peak splitting at slower scan rates (*v* ≤ 0.1 V s^–1^; representative examples in Figure S4). Split peaks are often observed on ferrocene-containing SAMs formed on electrodes, and they are typically attributed to intermolecular reactions between adjacent Fc molecules.^61,62^ It is unclear why only some of our samples display this behavior, but we suspect that inhomogeneities in the electrodeposition and/or SAM formation are the cause. We discuss nonideal responses in more detail below.

We also prepared SAMs on p^+^-Si/Au electrodes, which are highly doped and not photoactive (Figure S5 in the Supporting Information). The data in Figure S5 are qualitatively similar to Figure 4a, but the peaks are sharper and the currents are larger. We suspect that the differences between samples prepared with n-Si and p^+^-Si are caused by differences in the metal morphology of the two samples leading to changes in the interactions between FcHT molecules in the SAM. We used the difference in anodic peak position between the illuminated n-Si samples and the p^+^-Si samples to estimate the photovoltage (*V*_oc_) of the n-Si/Au/FcHT junctions, which is approximately 0.35 V. This value is consistent with our previous results using similar n-Si/Au photoelectrodes.^48,49^ Recently, we observed that samples have consistent photovoltage when *E*^0^ of the redox species is more positive than the potential of the valence band edge.^59^

We performed several control experiments to confirm that the observed voltammetry was due to the FcHT SAM attaching to the Au NPs. First, we performed CV at several different scan rates between 0.1 and 1.0 V s^–1^. Figure 4b shows a plot of anodic peak current versus scan rate current for n-Si/Au/FcHT samples. The relationship between the current and scan rate is linear (*R*^2^ = 0.997), confirming that the FcHT is attached to the surface.^63^ Next, we incubated freshly etched n-Si electrodes (without Au) in 1 mM FcHT and subsequently in 30 mM MCH. Figure S6a presents CVs of these electrodes in 0.1 M HClO_4_. The electrodes show a small peak around −0.2 V, suggesting that the thiol can react with the freshly etched Si surface, albeit to a much lesser extent than when Au is present (Figure S6b). Interestingly, the peak position for the FcHT on freshly etched n-Si is approximately 0.15 V more cathodic than observed on the n-Si/Au/FcHT samples, suggesting that *V*_oc_ is determined by the energy difference between the n-Si and FcHT in the absence of Au. Recent studies have shown that SAMs can form spontaneously between freshly etched Si and thiols (and disulfides).^51^ As an additional control experiment, we polarized freshly etched n-Si electrodes in the electrodeposition bath without the HAuCl_4_ and incubated them sequentially in FcHT and MCH. We observed only background redox activity after incubating the prepolarized electrodes in the thiol solutions (Figure S7). Next, we prepared SAMs containing only MCH on n-Si/Au. Figure S8 shows CVs of a n-Si/Au/MCH electrode in 0.1 M HClO_4_ in the presence and absence of illumination. These electrodes show almost no electrochemical activity, confirming that the supporting electrolyte and the backfilling MCH are not the cause of the observed voltammetry. Finally, Figure S9 shows CVs of n-Si/Au/FcHT electrodes in 0.1 M HClO_4_ before (Figure S9a) and after (Figure S9b) vortex mixing in DI water. We hypothesized that vigorous vortex mixing would dislodge any weakly bound FcHT molecules. Figure S9 shows only a small decrease in current after vortex mixing, suggesting that the SAM is strongly tethered to the Au surfaces.

To confirm that the FcHT is forming a monolayer on the surface, we determined the charge passed during oxidation by integrating the anodic scan of the CV and dividing by the scan rate. The surface coverage was determined using the following equation:
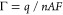
1where Γ is the surface coverage of the molecule (in mol cm^–2^), *q* is the charge passed during the oxidation wave (in C), *n* is the number of electrons transferred in the redox reaction (=1), *A* is the electrochemically active surface area (ECSA; in cm^2^), and *F* is Faraday’s constant (= 96,485 C mol^–1^). We employed ECSA because the electrodes consist of high surface area Au NPs, which increase the geometric area by ≈2–3 times, depending on the electrodeposition conditions (*vide infra*). ECSA was measured by performing illuminated CV in 0.5 M H_2_SO_4_ over the potential range from −0.2 to 1.6 V vs SCE and integrating the Au oxide reduction peak around 0.5 V on the cathodic sweep (see Figure S10 in the Supporting Information for example CVs in H_2_SO_4_). The charge under the oxide reduction peak was converted to surface area using the well-known conversion factor (= 390 μC cm^–2^ Au).^64^ We measured surface coverages of 4(±2) × 10^–10^ mol cm^–2^ (mean ± *s*; *n* = 3 independent samples). Assuming that Fc is close-packed with a diameter of 0.66 nm, the theoretical monolayer coverage for ferrocene on Au is ≈4.5 × 10^–10^ mol cm^–2^,^24^ which is in excellent agreement with our samples.

### Factors that Impact the Formation of FcHT SAMs on n-Si/Au Photoelectrodes

We next sought to understand how three preparation factors impact SAM formation and thus the observed electrochemical response. We investigated (1) the role of FcHT concentration for a fixed time, (2) the concentration of HAuCl_4_ in the electrodeposition bath, and (3) the incubation time at a fixed FcHT concentration.

#### Effect of the FcHT Concentration during Incubation

To determine how the concentration of FcHT impacted the electrochemical response, we prepared n-Si/Au/FcHT electrodes by incubating n-Si/Au electrodes in 1 or 2 mM solutions of FcHT for 60 min followed by a 90 min incubation in MCH. Figure S11 shows CVs of SAMs containing 1 mM FcHT (black) and 2 mM FcHT (red) tested in 0.1 M HClO_4_. CVs for samples prepared with 2 mm FcHT show a large increase in peak current compared with samples prepared with 1 mM FcHT. These samples also show evidence of a diffusional current (i.e., the current does not return to baseline at the anodic limit). The surface coverage of samples prepared with 2 mM FcHT is 2.2(±0.4) × 10^–9^ mol cm^–2^, approximately 5× larger than the films prepared with 1 mM FcHT. This surface coverage is also 5× larger than the theoretical monolayer coverage for Fc on Au, suggesting that multilayer films are formed under these conditions. Previous work has shown that self-assembled multilayers are formed for Fc-terminated alkanethiols when the concentration of the Fc-alkanethiols is elevated during incubation.^65,66^ Because higher FcHT concentrations produced multilayer films, we performed the remaining studies using 1 mM FcHT in the incubation solution.

#### Effect of HAuCl_4_ Concentration in the Electrodeposition Solution

Next, we investigated how the concentration of HAuCl_4_ in the electrodeposition solution influenced the voltammetry of the SAM. Note that in all previously discussed data, the concentration of HAuCl_4_ in the electrodeposition bath was 0.2 mM. Based on our previous work using n-Si/Pt electrodes, we suspected that changing the concentration of the metal precursor would change the morphology and loading of the metal NPs.^67^ Recently, Lazenby and co-workers showed that surface roughness and morphology of Au electrodes and microelectrodes had a dramatic impact on the performance of electrochemical aptamer-based sensors based on SAMs of DNA aptamers.^68^ We prepared electrodes by electrodepositing Au from an electrolyte containing 0.2, 0.5, or 1 mM HAuCl_4_ (along with 0.1 M K_2_SO_4_, 1 mM KCl, and 1 mM H_2_SO_4_) for 5 min at −1.945 V vs SCE. The electrodes were subsequently incubated sequentially in 1 mM FcHT for 60 min followed by 30 mM MCH for 90 min.

**Figure 5:**
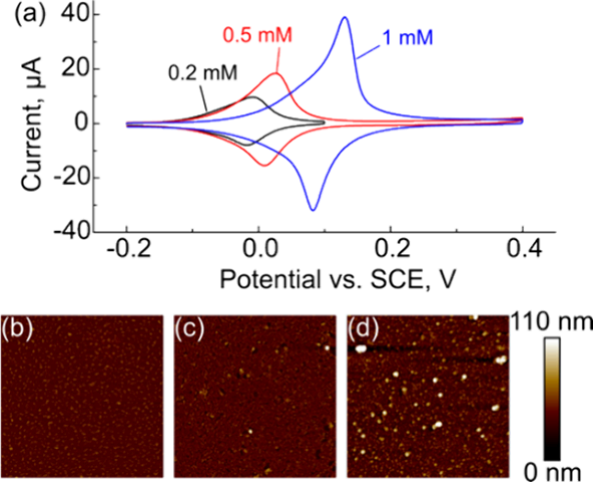
(a) Cyclic voltammograms of 1 mM FcHT monolayers in 0.1 M HClO_4_ with 0.2 mM, 0.5 mM, and 1 mM n-Si/Au NP LAES. AFM images (3 × 3 μm) of samples prepared with (a) 0.2 mM, (b) 0.5 mM, and (c) 1 mM HAuCl_4_ in the electrodeposition bath. Experimental conditions: *v* = 0.4 V s^–1^; reference = SCE; counter = glassy carbon rod.

Figure 5 shows representative CVs of samples prepared with 0.2 mM (black), 0.5 mM (red), and 1 mM (blue) HAuCl_4_. Qualitatively, we observe that the peak current, peak position, and peak separation all increase as the concentration increases. Representative figures of merit extracted from the data in Figure 5 are summarized in Table 2. As the concentration of HAuCl_4_ in the electrodeposition solution increases, we observe a statistically significant increase in ECSA between 0.2 and 0.5 mM/1 mM, but there was no difference in ECSA between 0.5 and 1 mM (Student’s *t* test, 95% confidence). This suggests that the surface is saturated with Au particles when 0.5 or 1 mM HAuCl_4_ is used in the electrodeposition bath. From each voltammogram, we calculated the surface coverage of Fc using eq 1 and found that all samples were consistent with monolayer formation (i.e., the true value of a monolayer, 4.5 × 10^–10^ mol cm^–2^, falls within the 95% confidence interval). However, the mean surface coverage for samples prepared with 1 mM HAuCl_4_ was ≈50% larger than the true value of a monolayer. We also observed a shift in *E*_1/2_ from 3(±11) to 82(±44) mV as the HAuCl_4_ concentration increased from 0.2 to 1 mM. We suspect that this shift is caused by the thickness of the Au layer increasing, leading to less light being absorbed by the semiconductor and in turn, decreased photovoltage.^60^

We observed an increase in the Δ*E*_p_ (from 12 ± 4 to 41 ± 16 mV) and a decrease in FWHM (from 89 ± 11 to 50 ± 22) as the gold concentration increased from 0.2 to 1 mM. Both changes suggest that the monolayers are becoming more disordered with increasing Au concentration. When samples are prepared with 0.2 mM HAuCl_4_ in the electrodeposition bath (as shown in Figures 4 and 5 and Table 2), the samples are nearly ideal, but at higher concentrations, they deviate significantly from ideal behavior.

**Table 2:** Summary of the CV and AFM Figures of Merit Obtained with n-Si/Au/FcHT Photoelectrodes^a^

**[Au] (mM)**	**ECSA (cm^2^)**	**Γ (**mol cm^–2^)	*E*_**1/2**_**(V) vs SCE**	**Δ***E***p (mV)**	**FWHM (mV)**	*d*_**particle**_**(nm)**	**RMS roughness (nm)**
0.2	0.14 ± 0.03	4(±2) × 10^–10^	0.003 ± 0.011	12 ± 4	89 ± 11	45 ± 10	6.6
0.5	0.20 ± 0.02	4(±4) × 10^–10^	0.029 ± 0.017	29 ± 11	86 ± 13	47 ± 12	10.4
1	0.20 ± 0.05	6(±4) × 10^–10^	0.082 ± 0.044	41 ± 16	50 ± 22	61 ± 26	10.6

a Values are reported as the mean ± standard deviation of three independently prepared samples from CVs recorded at 0.4 V s^–1^ in 0.1 M HClO_4_.

Comparing the data from Figure 5 with the CV in Figure 4, it is immediately clear that there are multiple redox environments present when higher concentrations of HAuCl_4_ are present in the electrodeposition bath. To qualitatively understand the behavior of these multiple redox environments, we deconvoluted the anodic traces of the three CVs present in Figure 5 using a Gaussian–Lorentzian peak fitting protocol described by Lee et al.^69^ Briefly, capacitance was subtracted from the data using a linear background subtraction and the data was fit with two peaks, a Gaussian peak at more cathodic potentials and a Lorentzian peak at more positive potentials. No limits were placed on peak widths, heights, or positions. Figure S12 shows the deconvoluted voltammograms along with the background subtracted experimental data and Table S1 presents the peak parameters for each subpeak. In all cases, we observed excellent agreement between the sum of the component peaks and the experimental data. Peak 1 is present at more cathodic potentials and has an FWHM of approximately 90 mV (range 87–99 mV). Peak 2 is present at more anodic potentials and has a narrower FWHM (range: 28–38 mV). In all three cases, peak 1 had a much larger area than peak 2 (2.3–3.6 × ). The fits show an anodic shift in the peak centers of each subpeak and an increase in peak current with increasing HAuCl_4_ concentration (also observable in Figure 5). We note that while deconvolution can provide some additional insight into the nature of the chemical environment, these results should not be viewed as quantitative. One significant drawback of this approach is that a fit for a given set of experimental data can converge with vastly different subpeaks. Figure S13 shows three examples of fits obtained for the same set of data with vastly differing subpeaks.

To further understand the photoelectrochemical data, we performed AFM topography imaging of the surfaces to characterize changes in morphology and roughness. Figure 5b, c, and d shows representative 3 μm by 3 μm AFM height images of n-Si/Au/FcHT samples prepared with 0.2, 0.5, and 1.0 mM HAuCl_4_ in the electrodeposition bath, respectively. As the concentration of the HAuCl_4_ in the electrodeposition solution increases, the morphology of the film becomes more disordered. At low concentrations, we observe a dense array of small particles. At higher concentrations, we observe larger particles and aggregates (>100 nm) and more clustering of the particles on the surface. From the AFM images, we determined the mean particle diameter and the RMS roughness of the surfaces (summarized in Table 2). The mean particle diameter increased from 45 ± 10 to 61 ± 26 nm and the RMS roughness increased from 6.6 to 10.6 nm as the HAuCl_4_ concentration increased from 0.2 to 1.0 mM, respectively. The standard deviation in the particle size (10 and 26 nm for 0.2 and 1.0 mM HAuCl_4_, respectively) and RMS roughness both suggest that the surfaces are becoming more disordered with increasing HAuCl_4_ concentration.

#### Effect of FcHT Incubation Time

**Figure 6:**
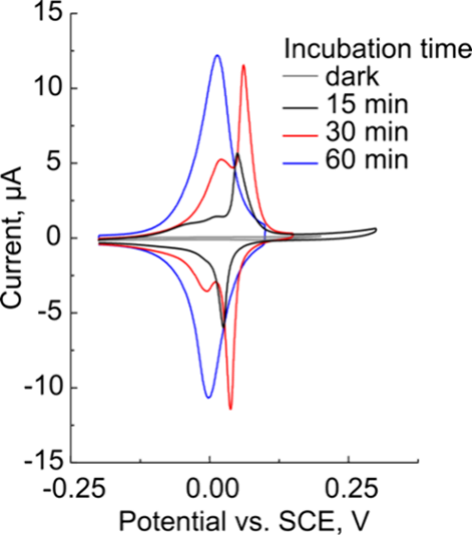
CVs of n-Si/Au/FcHT electrodes prepared by incubating in 1 mM FcHT solutions for 15 (black trace), 30 (red trace), or 60 min (blue trace). A representative dark trace is also shown in gray. Electrolyte: 0.1 M HClO_4_; reference: SCE; counter: glassy carbon rod; *v* = 0.4 V s^–1^.

We next investigated how the incubation time impacted the voltammetry of the n-Si/Au/FcHT photoelectrodes. In these experiments, we incubated the n-Si/Au electrodes in aqueous solutions of 1 mM FcHT for 15, 30, or 60 min followed by a 90 min incubation in 30 mM MCH. Representative CVs for these samples are shown in Figure 6. For samples incubated for 15 min (black trace), there are three redox signals apparent: two irreversible waves at −0.04 and 0.01 V and a quasi-reversible oxidation peak at 0.05 V vs SCE. The peak at 0.05 V has a height of approximately 5.6 μA and a Δ*E*_p_ of approximately 25 mV. After 30 min (red trace), the two waves at −0.4 and 0.01 V combine to form a quasi-reversible peak around 0.02 V with a second larger peak at 0.06 V. Both anodic peaks have corresponding reduction peaks with similar peak separations (Δ*E*_p_ ≈ 24 and ≈23 mV for 0.02 and 0.06 V peaks, respectively). After 60 min (blue trace), only a single quasi-reversible peak is evident at 0.01 V with Δ*E*_p_ ≈ 14 mV. As the FcHT incubation time increases from 15 to 60 min, the FcHT surface coverage (Γ) increases from 1.3 × 10^–10^ to 4.5 × 10^–10^ mol cm^–2^, demonstrating that the growth of the monolayer is incomplete at short times. These data present a “snap shot” of the growth and organization of the FcHT SAMs and suggest that the FcHT requires significant time to form an organized monolayer. At shorter time scales, multiple peaks are observed, suggesting different chemical environments for the surface-bound Fc moieties. However, after 60 min, the monolayer is sufficiently organized to yield a single redox peak. As previously mentioned, ≈1/3 of the samples prepared with optimized conditions (0.2 mM HAuCl_4_ in the electrodeposition solution and 1 mM FcHT incubated for 60 min) still exhibit multiple peaks. It is plausible that these samples may have required additional time for the SAM to organize.

We performed peak fitting analysis of the data in Figure 6 (*vide supra*) where a Gaussian–Lorentzian fit of the data was used to qualitatively understand the changes in each subpeak.^69^ We obtained suitable fits of the experimental data using three peaks for the 15 and 30 min incubation times, but only two peaks for the 60 min incubation time. Fits of the data and a summary of the results are presented in Figure S14 and Table S2, respectively.

#### Summary of Preparation Effects

Taken together, the data in Figures S5, S6, and S10 suggest that the chemical environment of the Fc moiety is dramatically impacted by preparation conditions. As shown in Figures 2 and 5, our samples are composed of Au NPs, with a heterogeneous distribution of size and shape, which are randomly dispersed on the surface of Si (100). Perhaps unsurprisingly, this disorder can lead to nonideal voltammetry—especially when higher concentrations of HAuCl_4_ and shorter FcHT incubation times are used to prepare the SAM. Previously, Nijhuis and co-workers performed detailed spectroscopic and electrochemical measurements along with molecular dynamics simulations to untangle how disorder in Fc-containing SAMs on metallic Au impacted the electrochemistry.^61^ Their findings (and others’)^69^ suggest that for SAMs where Fc is separated from the Au surface by six methylene groups (the case for this study), multiple redox peaks originate from interactions between Fc and the electrolyte, Fc and other Fc molecules, and Fc buried in the SAM (and thus shielded from the electrolyte). We suspect that for the rough, disordered surfaces used herein, the SAM is likely to be less well-ordered, leading to more intermolecular interactions between Fc moieties. This hypothesis is supported by the work of Landis and co-workers, who investigated redox-active SAM formation on nanoporous Au, finding that changes in the porosity of the nanoporous Au had substantial effects on the intermolecular interactions between Fc molecules in the SAMs.^62^ Both of these studies were performed with metallic Au electrodes, and therefore, no photoeffects were considered. In the samples prepared here, variations in the photovoltage are expected due to variations in Au nanoparticle size. The Boettcher group has characterized such variations in photovoltage on other semiconductor/metal systems for water-splitting applications.^70^ Ciampi and co-workers observed that for Fc-containing SAMs on Si, nonideal behavior (multiple peaks and/or FWHM not equal to ≈90 mV) originates from variations in the photovoltage across the semiconductor surface.^71^ Interestingly, Ciampi’s group observed that peak splitting was dependent on illumination intensity (*vida infra*).

We hypothesize that the heterogeneity of the Au layer can contribute to nonideal behavior in two ways: first, by contributing to the disorder in the SAM as discussed by Nijhuis et al.,^61^ Landis et al.,^62^ Lee et al.,^69^ and Dempsey et al.^72^ Second, the distribution of NP sizes is also likely to affect *V*_oc_ across the semiconductor/metal junction.^70,73,74^ We explore this in more detail below.

### Impact of Light Intensity on n-Si/Au/FcHT Electrode Behavior

Recent work has shown that the illumination intensity impacts the kinetics and photovoltage of ferrocene-containing redox-active SAMs attached directly to n-Si. Gooding and co-workers showed that n-Si modified with monolayers of Fc displayed a cathodic shift of ≈160 mV under illumination intensities ranging from ≈3 to 90 mW cm^–2^.^28^ Concurrently, the standard electron transfer rate constant increased by a factor of ≈3 with the same range of intensities. The number of photogenerated holes is thought to directly influence the rate of the reaction in two ways: first, oxidation is limited by the number of available holes, and increasing the intensity increases the number of holes. Second, the driving force for hole transfer from Si to Fc is increased with increasing intensity. More recently, Ciampi and co-workers observed peak splitting using similar n-Si/Fc electrodes illuminated at low intensities (≈1 mW cm^–2^). This peak splitting was attributed to variations in the local photovoltage due to the n-Si surface and was not present at illumination intensities >10 mW cm^–2^.^71^ A fundamental difference between those previous studies and those herein is that to oxidize ferrocene, holes must first be transferred from Si to Au before being transferred to Fc. This experimental arrangement decouples the solid-state interfacial charge transfer from the molecular HET.

**Figure 7:**
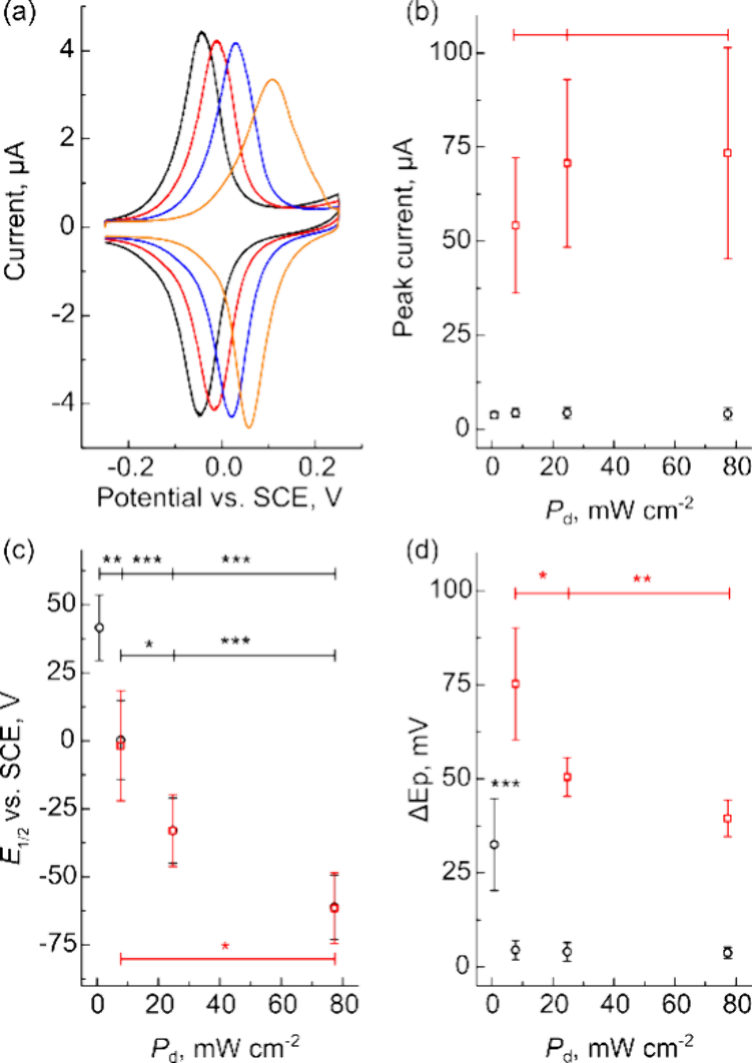
Changing the illumination intensity has an impact on the kinetics of HET reactions on n-Si/Au/FcHT electrodes. (a) CVs of n-Si/Au/FcHT in 0.1 M HClO_4_ collected with 0.81 (orange trace), 7.8 (blue trace), 25 (red trace), and 77 (black trace) mW cm^–2^ white light. CVs were recorded at 0.25 V s^–1^ in an electrolyte containing 0.1 M HClO_4_. Plots showing the effect of illumination intensity on (b) peak current, (c) peak position (*E*_1/2_), and (d) peak potential separation (Δ*E*_p_). *p < 0.05; **p < 0.01; ***p < 0.001.

To understand how illumination intensity impacts the kinetics of the n-Si/Au/FcHT samples, we performed CV at scan rates between 0.25 and 25 V s^–1^ under illumination intensities ranging from 0.81 to 77 mW cm^–2^. Based on the results of the optimization experiments described above, we prepared samples by electrodepositing Au onto freshly etched n-Si from a 0.2 mM HAuCl_4_ solution and subsequently incubating the electrodes in 1 mM FcHT solutions for 60 min followed by a 90 min incubation in MCH. Figure 7a shows CVs of n-Si/Au/FcHT electrodes in 0.1 M HClO_4_ acquired using 0.81, 7.8, 25, and 77 mW cm^–2^ light at 0.25 V s^–1^. CVs collected at various scan rates for the four different intensities are presented in Figure S15 in the Supporting Information. Qualitatively, decreasing the illumination intensity shifted the voltammograms toward more anodic potentials, increased Δ*E*_p_, and broadened the FWHM of the voltammograms. Figure 7b shows the effect of illumination intensity on the peak current for 0.25 V s^–1^ (black circles) and 25 V s^–1^ (red squares). For each scan rate, there was no statistical difference between the means (*p* > 0.05; one-way ANOVA with Bonferroni correction). This behavior was expected because the magnitude of the peak is governed by the amount of FcHT adsorbed to the Au surfaces, in addition to the nature of the kinetics of the electrochemical reaction (i.e., reversible redox waves will have larger peaks than quasi-reversible or irreversible waves). At the lowest illumination intensities, we observed that the anodic traces became limited by light when probed at higher scan rates (Figure S15c,d).

Figure 7c shows that the peak position (*E*_1/2_) shifts toward more cathodic potentials with increasing intensity, consistent with results from the Gooding and Ciampi groups.^28,71^ There was no change in *E*_1/2_ with the scan rate. The shift in peak position is caused by changes in *V*_oc_ of the n-Si/Au/FcHT electrodes; as the illumination intensity increases, more carriers are generated in the semiconductor, which changes the splitting of the electron and hole quasi-Fermi levels.^60^ The quasi-Fermi level splitting will be increased with increasing intensity, leading to larger *V*_oc_. The magnitude of the change in *V*_oc_ is approximately 100 mV over the range of intensities studied here. The oxidation peaks for the highest (77 mW cm^–2^; black trace) and lowest intensities (0.81 mW cm^–2^; orange trace) are separated along the potential axis. One possible application of this observation is the opportunity to use light intensity to multiplex electrochemical sensors with identical redox tags, although more thorough investigations are required.

Figure 7d shows the impact of illumination intensity on Δ*E*_p_, which is related to the electron transfer rate. At 0.25 V s^–1^_,_ Δ*E*_p_ is consistent between ≈8 and 77 mW cm^–2^ but broadens significantly at 0.8 mW cm^–2^. At 25 V s^–1^, Δ*E*_p_ increases significantly between 8 and 77 mW cm^–2^ (note that above 1 V s^–1^, a limiting current was observed on the anodic trace under 0.8 mW cm^–2^ illumination). These data demonstrate that the electron transfer kinetics are impacted by the illumination intensity, with higher intensities leading to faster kinetics. However, these effects are most pronounced when higher scan rates are used. We suspect that higher scan rates are required to observe these effects with n-Si/Au/FcHT photoelectrodes because both the charge transfer between n-Si/Au and the electrochemical kinetics of FcHT oxidation on Au are each extremely fast.

As previously mentioned, Ciampi and co-workers observed peak splitting at low illumination intensities that were attributed to photovoltage variations across the interface.^71^ While we did not observe peak splitting, we did observe peak broadening with decreasing intensity. The FWHM of samples increased from 92 to 134 mV as the illumination intensity decreased from 77 to 0.81 mW cm^–2^. Given that the SAM is unlikely to be reorganized on the time scale of the experiments (∼2 s), it is plausible that the broadening we observe may be caused by variations in *V*_oc_ across the surface. This scenario seems especially likely given the distribution of Au particle sizes on these samples (Figure 2).

## Conclusions

Here, we investigated the photoelectrochemistry of redox-active SAMs on semiconductor/metal junctions, where the chemical modification occurs at the metal site instead of the semiconductor. We prepared n-Si/Au photoelectrodes using a benchtop electrodeposition procedure and subsequently formed mixed SAMs consisting of FcHT and MCH by sequential incubation in aqueous solutions of each thiol. We investigated how three preparation factors (FcHT concentration, FcHT incubation time, and Au electrodeposition precursor concentration) impact SAM formation and thus the observed electrochemical response. We characterized the sensors using physical and electrochemical methods, finding that under certain conditions, nearly ideal cyclic voltammetry for a surface-confined redox molecule was obtained. We also investigated the effect of illumination intensity on the observed response, finding that higher illumination intensity improves the HET kinetics for FcHT oxidation.

Under certain conditions (e.g., when the HAuCl_4_ or FcHT concentrations are high or when the incubation time is <1 h), we observed irregular voltammetry characterized by multiple redox peaks, which originate from variations in the chemical environment(s) of the surface-bound ferrocene moieties. We suspect the differences come from variations in the SAM ordering,^61^ Fc–Fc interactions,^62^ and photovoltage.^71^ Unfortunately, the measurements presented here do not definitively suggest one mechanism over another and these samples will require more careful study with scanning probe techniques to characterize the relative importance of each variable. These data highlight how caution should be exercised when fabricating these types of sensors, because under many conditions the voltammetry is not ideal.

The measurements presented herein are the first to demonstrate the photoelectrochemistry of a semiconductor/metal/molecule interface. The results are significant because they open up new avenues for future inquiry. First, because the interfaces have excellent photoelectrochemical behavior, they can be used as a test bed for future studies of electron transfer at semiconductor/metal interfaces. While this study investigated a relatively simple redox system (FcHT/MCH), there is considerable scope for expansion to probe the effect of linker length, counterion, redox mediator, etc. Second, the formation of the SAM using Au–S chemistry can be extended to other systems including biomolecules, catalysts, etc., which may enable new applications in semiconductor/metal photoelectrochemistry.

## Supporting Information Available

Additional XPS scans; electrochemical impedance spectroscopy (EIS) characterization; additional CV data and control experiments; deconvolution of redox waves containing multiple redox environments; and additional CVs of variable light intensity data (PDF)
