# Supplementary material for: Photoelectrochemistry of Redox-Active Self-Assembled Monolayers Formed on n-Si/Au Nanoparticle Photoelectrodes
Source: Langmuir. 2024 Aug 7;40(33):17536–46. doi: 10.1021/acs.langmuir.4c01751 (PMC11340028; doi:10.1021/acs.langmuir.4c01751)
Supplement: Supplementary file 1 — la4c01751_si_001.pdf [file la4c01751_si_001.pdf]

Electronic supporting information for

Photoelectrochemistry of redox-active self-assembled monolayers formed on n-Si/Au nanoparticle photoelectrodes

Kayla M. Mancini,[†] Yousef Khatib,[†] Lauren Shahine,[†] and Glen D. O'Neil[†‡*]

[†]Department of Chemistry and Biochemistry, Montclair State University, Montclair, NJ 07043

[‡]Sokol Institute for Pharmaceutical Life Sciences, Montclair State University, Montclair, NJ 07043

*email: oneilg@montclair.edu; phone: +1 (973) 655-3791

Table of contents

Section S1. Additional XPS scans…………………………………………………………………... S2
Section S2. Electrochemical impedance spectroscopy (EIS) characterization ……………………………S3
Section S3. Additional CV data and control experiments …………………………………………………S4
Section S4. Deconvolution of redox waves containing multiple redox environments ……………………S8
Section S5. Additional CVs of variable light intensity data ……………………………………………S11

Section S1. Additional XPS scans

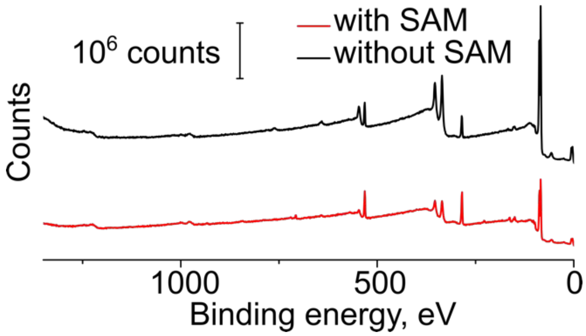

Figure S1. XPS survey scans for (black trace) n-Si/Au and (red trace) n-Si/Au/Fc-SAM electrodes.

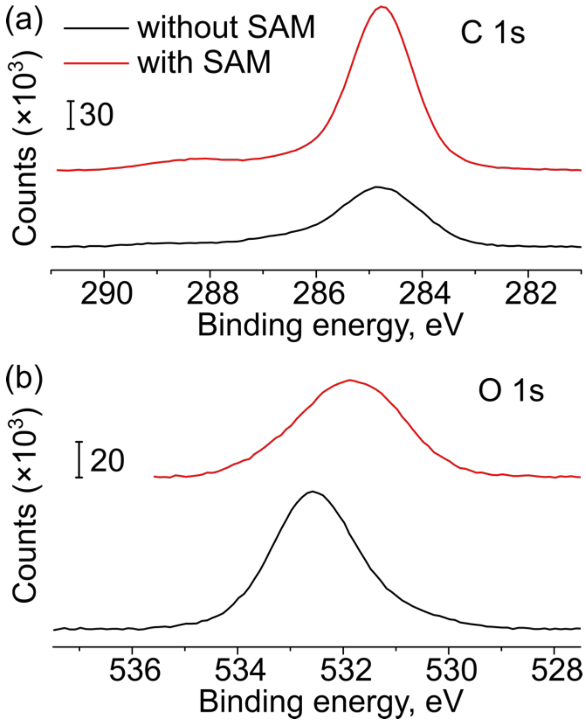

Figure S2. High-resolution XPS (a) C 1s and (b) O 1s scans for (black trace) n-Si/Au and (red trace) n-Si/Au/FcHT electrodes.

We estimated the thickness of the SAM to be ≈30 Å using Bain and Whitesides's method:[1]

$$I_{SAM} = I_{Au} e^{-d_{SAM}/\lambda_{SAM}} \quad \text{(S1)}$$

where I_{SAM} and I_{Au} are the intensities of the Au $4f_{5/2}$ peak, d_{SAM} is the thickness of the SAM (in Å), and λ_{SAM} is the attenuation wavelength (42 Å).[1,2] This is considerably thicker than we expected for a monolayer with a six-carbon chain. However, Bain and Whitesides's method assumes that the surface is flat.[1] In our samples, the electrodeposited Au nanoparticles introduce significant roughness to the surface (Figure 2), leading to an underestimation of the attenuation wavelength and an overestimation of the SAM thickness.[1]

Section S2. Electrochemical impedance spectroscopy (EIS) characterization

Figure S2 shows reciprocal square capacitance versus voltage plots for n-Si/Au/Fc-SAM recorded in a 0.1 M $HClO_4$ electrolyte over a –1 to 0 V range with an impedance measurement made every 10 mV. The electrochemical cell was housed inside a Thorlabs dark box. Equation S1 relates the capacitance of the space charge region (C_{SC}) to the potential of an electrode versus a reference (E):[3]

$$\frac{1}{C_{SC}^2} = \frac{2}{q\,\varepsilon\,\varepsilon_0\,N_d\,A^2}\left(E - E_{fb} - \frac{k_B T}{q}\right) \tag{S2}$$

where C_{sc} is the space charge capacitance (in F), q is the fundamental charge on an electron (=1.6•10^{-19} C), ε is the dielectric constant of the semiconductor (11.7 for Si),[4] ε_0 is the permittivity of free space, N_d is the bulk dopant carrier concentration (in cm^{-3}), A is the electrode area (=0.071 cm^2), E is the applied potential (in V), E_{fb} is the flat band potential (in V), k_B is Boltzmann's constant, and T is the absolute temperature (in K). We calculated the space charge capacitance using the imaginary component of the impedance and equation S3:

$$C_{sc} = (2\pi v Z")^{-1} \tag{S3}$$

where v is the frequency (in Hz), and Z' is the imaginary component of the impedance (in Ω). We fit the data to the Mott-Schottky equation to find the flat-band potential and the bulk dopant concentration. The x-intercept of the linear portion of the the C^{-2} vs. E plot corresponds to $E_{fb} + k_B T q^{-1}$, while the slope of the line is related to bulk dopant concentration, N_d:

$$slope = \frac{2}{q\,\varepsilon\,\varepsilon_0\,N_d\,A^2} \tag{S4}$$

The conduction band edge (E_{cb}) can be estimated using equation S5:

$$E_{cb} = E_{fb} + k_B T ln\left(\frac{N_d}{N_c}\right) \tag{S5}$$

where N_c is the effective density of states of the conduction band for Si (=2.8•10^{19}).[4] Finally, the valence band edge (E_{vb}) is calculated by adding the band gap energy (E_g; =1.1 eV for Si) to the conduction band edge.

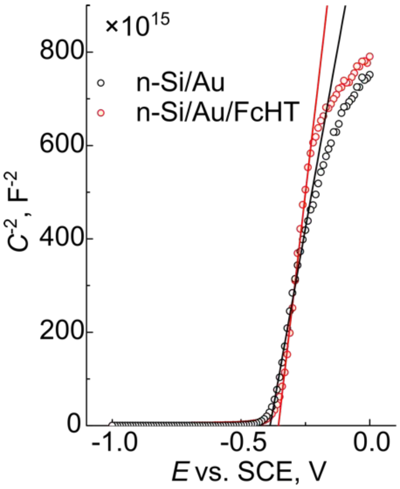

Figure S3. Reciprocal square capacitance plots of n-Si/Au and n-Si/Au/FcHT-SAM electrodes in the dark in 0.1 M $HClO_4$. The solid lines represent the linear regression of the linear portion of the plot – in both cases R^2 > 0.99; frequency = 50 kHz, amplitude = 5 mV, E_{step} = 10 mV; reference = SCE, counter = glassy carbon rod.

Section S3. Additional CV data and control experiments

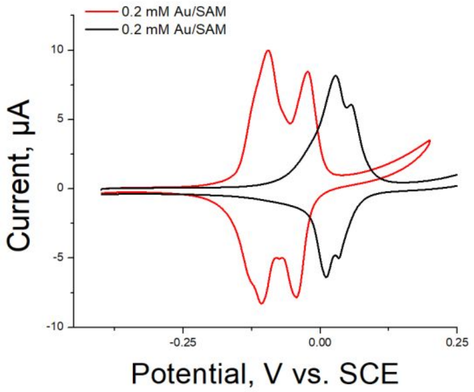

Figure S4. Examples of CVs showing peak splitting for n-Si/Au/FcHT photoelectrodes in 0.1 M $HClO_4$ at 0.1 V s^{-1}. Note these measurements were performed using two different light sources with differing intensities.

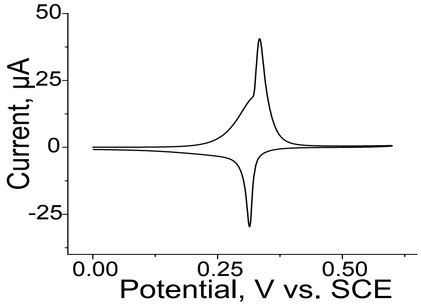

Figure S5. CVs of p$^+$-Si/Au/FcHT-SAM electrodes in 0.1 M $HClO_4$ at 0.1 V s^{-1}. Reference = SCE, counter = glassy carbon rod

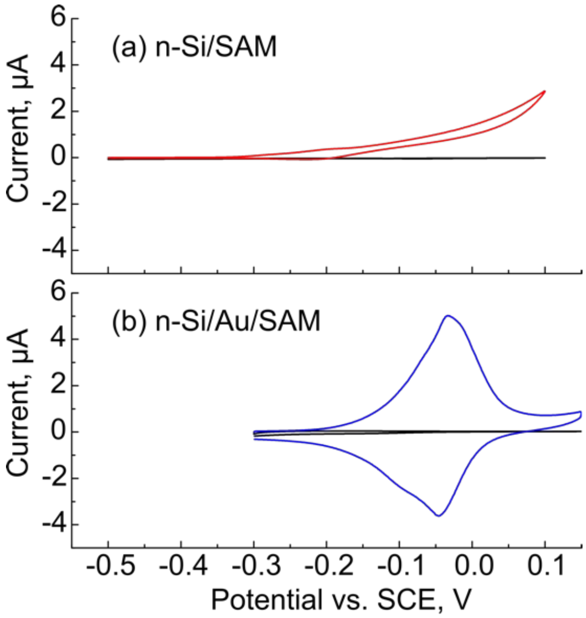

Figure S6. CVs of n-Si/FcHT-SAM electrodes in 0.1 M $HClO_4$ at 0.1 V s^{-1}; black trace = dark current; reference = SCE, counter = glassy carbon rod.

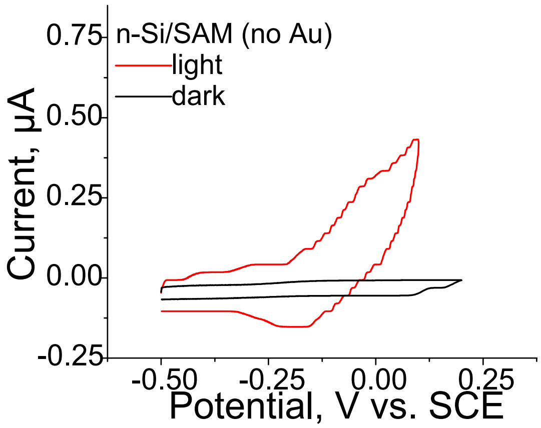

Figure S7. CVs of pre-polarized n-Si/FcHT photoelectrodes in 0.1 M $HClO_4$ at 0.1 V s^{-1}.

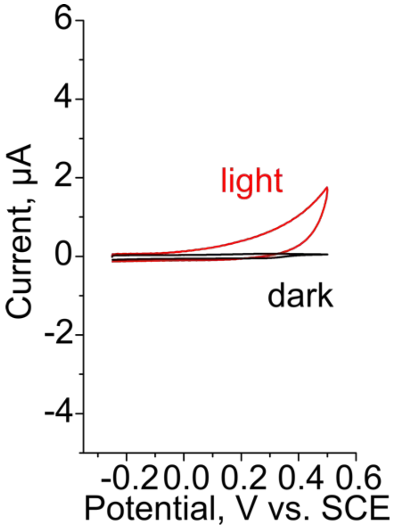

Figure S8. CVs of n-Si/Au/MCH-SAM electrodes in 0.1 M $HClO_4$ at 0.1 V s^{-1} in the dark (black trace) and illuminated (red trace). Reference = SCE, counter = glassy carbon rod.

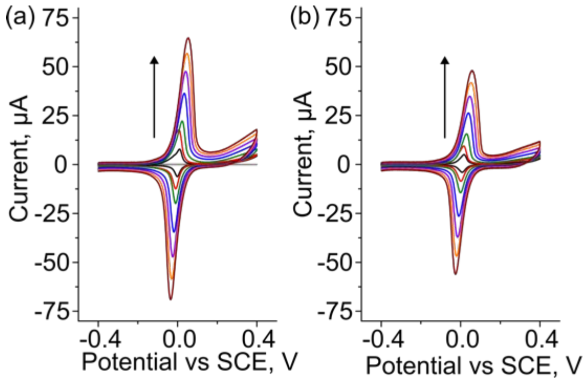

Figure S9. CVs of n-Si/Au/FcHT-SAM electrodes in 0.1 M $HClO_4$ at scan rates between 0.1 and 1.0 V s^{-1} (a) before and (b) after 1 minute of vortex mixing. Arrow indicates increasing scan rates. Reference = SCE, counter = glassy carbon rod.

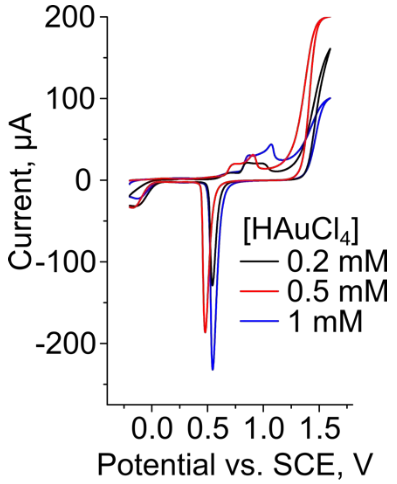

Figure S10. CVs of n-Si/Au electrodes in 0.5 M H_2SO_4 at 0.1 V s^{-1} prepared with 0.2 mM (black trace), 0.5 mM (red trace), and 1 mM (blue trace) $HAuCl_4$ in the electrodeposition bath. Reference = SCE, counter = glassy carbon rod.

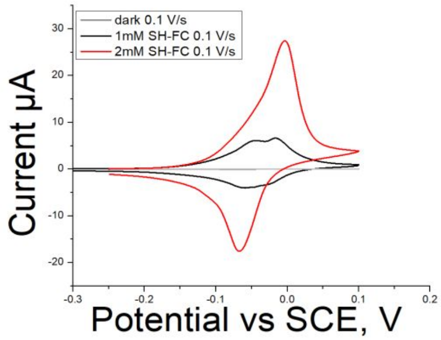

Figure S11. CVs of n-Si/Au/FcHT photoelectrodes prepared by incubating in aqueous 1 mM (black trace) and 2 mM (red trace) FcHT solutions for 60 minutes; Reference = SCE, counter = glassy carbon rod.

Section S4. Deconvolution of redox waves containing multiple redox environments.

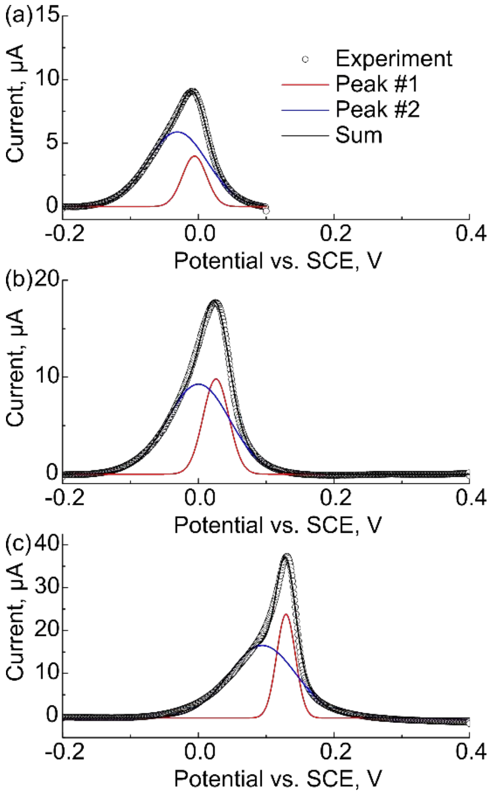

Figure S12. Deconvoluted anodic traces of anodic portions of CVs that are presented in Figure 5 of the main text. n-Si/Au/FcHT samples were prepared with (a) 0.2, (b) 0.5, or (c) 1 mM $HAuCl_4$ in the electrodeposition bath and incubated in a 1 mM FcHT solution for 60 minutes. Symbols are the raw data, the red and blue traces are individual components and the black trace is the sum of the fits.

Table S1. Summary of peak fitting parameters from Figure S12.

**$HAuCl_4$, mM**	**Peak 1**			**Peak 2**			**Peak 1/Peak 2 ratio**
	$E_{1/2}$, V vs. SCE	fwhm, V	height, μA	$E_{1/2}$, V vs. SCE	fwhm, V	height, μA	
0.2	-0.031	0.087	5.9	-0.006	0.036	4.0	3.6
0.5	0.000	0.092	9.3	0.026	0.038	9.8	2.3
1	0.094	0.099	16.9	0.129	0.028	24.2	2.5

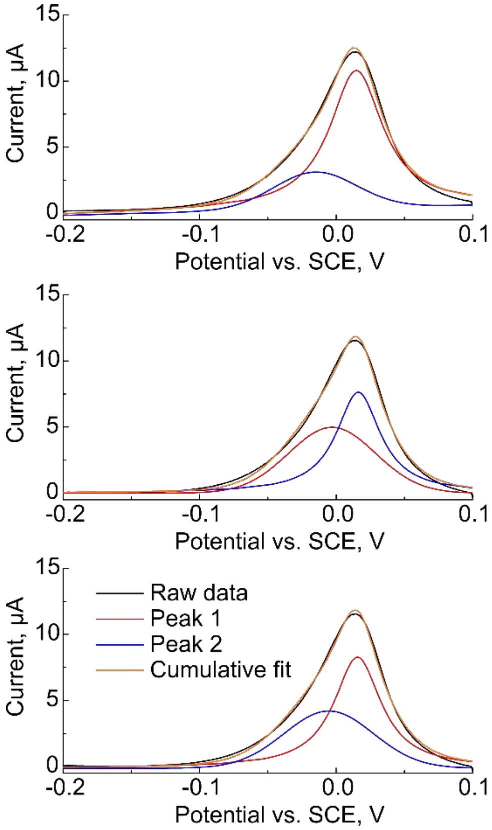

Figure S13. Examples of non-unique fits of the same experimental data.

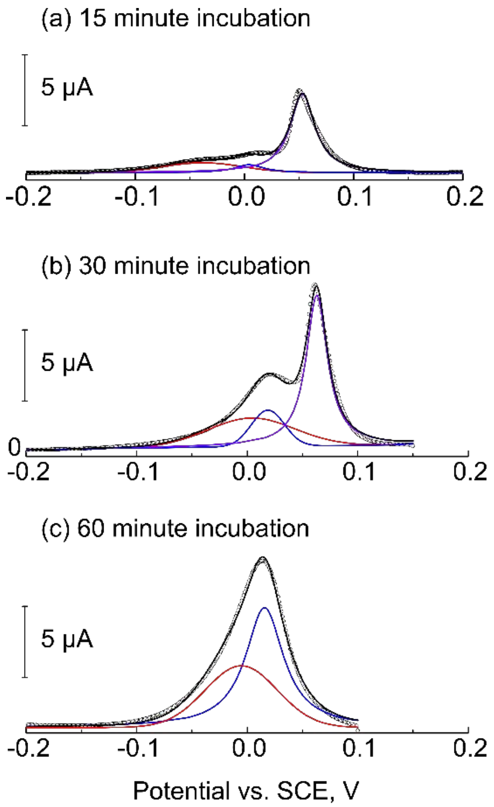

Figure S14. Deconvolution of the anodic portion of the CVs that are presented in Figure 6 of the main text. N-Si/Au/FcHT samples were prepared with 0.2 mM $HAuCl_4$ in the electrodeposition bath and incubated in 1 mM FcHT solutions for (a) 15, (b) 30, or (c) 60 minutes. Symbols are the raw data, the red, blue, and purple traces are individual components and the black trace is the sum of the fits.

Table S2. Summary of fit fitting parameters from Figure S13.

t_{inc}, min	Peak 1				Peak 2				Peak 3			
	$E_{1/2}$, V	fwhm, V	height, μA	Area, %	$E_{1/2}$, V	fwhm, V	height, μA	Area, %	$E_{1/2}$, V	fwhm, V	height, μA	Area, %
15	-0.038	0.086	0.67	19.2	0.004	0.032	0.53	8.5	0.053	0.028	5.28	72.3
30	0.004	0.093	2.00	29.1	0.019	0.035	2.54	14.0	0.063	0.023	10.52	56.9
60	-0.005	0.077	4.33	64.7	0.016	0.042	8.39	35.3	No peak			

Section S5. Additional CVs of variable light intensity data

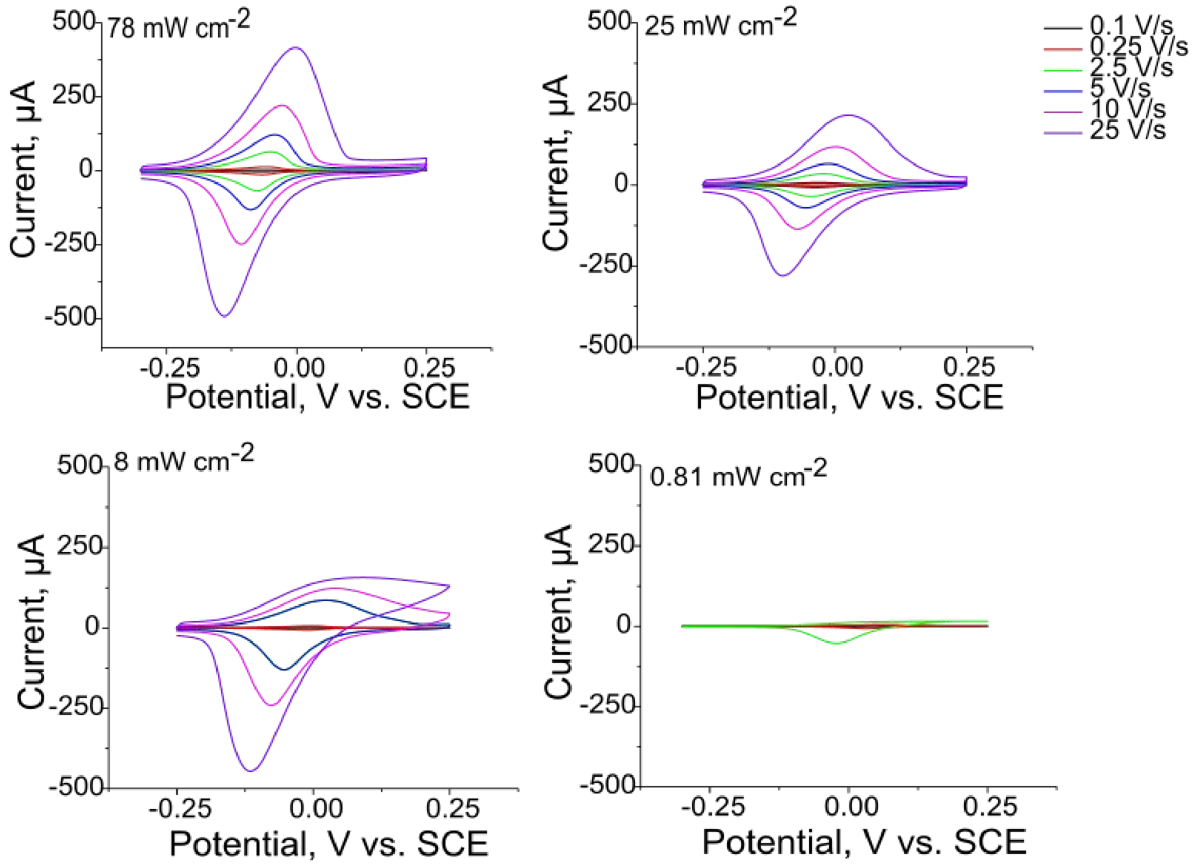

Figure S15. CVs of n-Si/Au/FcHT photoelectrodes in 0.1 M $HClO_4$ illuminated collected at scan rates between 0.1 and 25 V s^{-1} and illuminated with (a) 77, (b) 25, (c) 8, and (d) 0.81 mW cm^{-2} white light.

Section S6. References

(1) Bain, C. D.; Whitesides, G. M. Attenuation Lengths of Photoelectrons in Hydrocarbon Films. *J. Phys. Chem.* **1989**, *93* (4), 1670–1673. https://doi.org/10.1021/j100341a095.

(2) Wong, R. A.; Yokota, Y.; Wakisaka, M.; Inukai, J.; Kim, Y. Discerning the Redox-Dependent Electronic and Interfacial Structures in Electroactive Self-Assembled Monolayers. *Journal of the American Chemical Society* **2018**, *140*, jacs.8b05885. https://doi.org/10.1021/jacs.8b05885.

(3) Gelderman, K.; Lee, L.; Donne, S. W. Flat-Band Potential of a Semiconductor: Using the Mott–Schottky Equation. *Journal of Chemical Education* **2007**, *84* (4), 685. https://doi.org/10.1021/ed084p685.

(4) Acharya, S.; Lancaster, M.; Maldonado, S. Semiconductor Ultramicroelectrodes: Platforms for Studying Charge-Transfer Processes at Semiconductor/Liquid Interfaces. *Analytical Chemistry* **2018**, *90*, 12261–12269. https://doi.org/10.1021/acs.analchem.8b03574.
